# The Interaction and Effect of a Small MitoBlock Library as Inhibitor of ALR Protein–Protein Interaction Pathway

**DOI:** 10.3390/ijms25021174

**Published:** 2024-01-18

**Authors:** Riccardo Muzzioli, Angelo Gallo

**Affiliations:** CERM, University of Florence, Via L Sacconi 9, 50019 Sesto Fiorentino, Italy; rmuzzioli@solvetx.com

**Keywords:** NMR spectroscopy, drug screening, mitochondrial import pathway

## Abstract

MIA40 and ALR of the MIA pathway mediate the import of protein precursors that form disulfides into the mitochondrial intermembrane space. This import pathway is suggested to be a linear pathway in which MIA40 first binds to the precursor via a disulfide linkage and oxidizes it. Subsequently, ALR re-oxidizes MIA40 and then ALR transfers electrons to terminal electron acceptors. However, the precise mechanism by which ALR and MIA40 coordinate translocation is unknown. With a collection of small molecule modulators (MB-5 to MB-9 and MB-13) that inhibit ALR activity, we characterized the import mechanism in mitochondria. NMR studies show that most of the compounds bind to a similar region in ALR. Mechanistic studies with small molecules demonstrate that treatment with compound MB-6 locks the precursor in a state bound to MIA40, blocking re-oxidation of MIA40 by ALR. Thus, small molecules that target a similar region in ALR alter the dynamics of the MIA import pathway differently, resulting in a set of probes that are useful for studying the catalysis of the redox-regulated import pathway in model systems.

## 1. Introduction

Various cellular compartments, such as the chloroplast, endoplasmic reticulum, the bacterial periplasmic space, and the mitochondrial intermembrane space (IMS), depend on redox chemistry for protein translocation and folding [1,2,3,4]. Although these pathways exhibit shared characteristics involving oxidoreductases and terminal electron acceptors, it is important to note that the specific proteins involved are not conserved. MIA40/Mia40 and ALR/Erv1 (MIA40/Mia40 and ALR/Erv1, with capital letters referring to human protein, lowercase letters referring to yeast protein or more in general not human proteins; Mitochondrial Import and Assembly protein 40-MIA40 and Augmented Liver Regeneration-ALR, Essential for respiration and vegetative growth protein 1-Erv1) are key and essential protein components of the mitochondrial import and oxidative folding machinery in the IMS in various organisms. They are essential for ensuring the correct biogenesis of many IMS proteins, as well as for maintaining the redox balance within the mitochondria. These proteins are fundamental for mitochondrial function and overall cellular health.

In detail, these two proteins, MIA40 and ALR, constitute, in mitochondria, the Mitochondrial intermembrane space Import and Assembly (MIA) pathway and their specific role in this pathway entails the oxidoreductase (MIA40/Mia40) and the sulfhydryl oxidase (ALR/Erv1) activities [5,6,7,8] with the corresponding MIA40/CHCHD4 (coiled-coil-helix-coiled-coil-helix domain containing 4) and ALR/GFER (Growth Factor Erv1) in higher eukaryotes, respectively [9,10]. As the imported protein precursor passes through the Translocase of the Outer Membrane (TOM) complex, MIA40 binds to the unfolded precursor via a disulfide linkage and locks the unfolded protein in the IMS [11,12,13,14]. The precursor with the correct fold and insertion of disulfide bonds is subsequently released from the reduced form of MIA40; then, ALR re-oxidizes MIA40, priming for another round of import. Reduced ALR transfers electrons to oxygen or cytochrome c and the electron transport chain [8,15,16], thereby re-oxidizing ALR.

Precursors that use the MIA pathway typically acquire disulfide bonds to reach the correct fold to function. Examples are the small TIM (translocase of the inner membrane) proteins with a twin CX3C motif in the TIM22 import pathways [17], a group with a twin CX9C motif that regulates assembly of the respiratory complexes [18], and additional substrates with alternative cysteine motifs [19]. In addition to CX3C and CX9C protein groups, the MIA pathway also mediates the import of two precursors into the matrix: APE1 (Human AP endonuclease 1), involved in the mitochondrial DNA repair [20], and mitochondrial ribosomal protein Mrp10 [21].

In human cells, MIA substrates have been linked to neurodegenerative diseases such as CHCHD10 (coiled-coil-helix-coiled-coil-helix domain containing 10) in amyotrophic lateral sclerosis [22] and CHCHD2 (coiled-coil-helix-coiled-coil-helix domain containing 2) in Parkinson’s Disease [23]. Tumor suppressor p53 localization to mitochondria has also been shown to depend on the MIA pathway [24]. Consequently, the MIA pathway governs the import of a diverse range of precursors, with some of them having implications for degenerative diseases. In this case, NMR spectroscopy has provided insight into the molecular mechanisms of the MIA pathway. MIA40 contains a redox-active CPC motif that functions as a binding platform in which a transient disulfide bond is formed with a cysteine residue in the internal IMS Targeting Signal (ITS) or mitochondrial intermembrane space sorting (MISS) motif on the precursor that has been translocated into the IMS [14,19,25,26]. The CPC motif is oxidized as electrons are transferred from the precursor, generating an oxidized precursor, and facilitating precursor folding [27]. MIA40 also contains two additional CX9C motifs that have a structural role [14], but also may accept electrons if more than two electrons are being transferred [28].

ALR is a protein that assembles as a homodimeric complex and contains two distinct CXXC motifs that are central for disulfide exchange reactions [29]. The first CXXC motif, referred to as the shuttle CXXC, is located within the N-terminal 80 amino acids in an unstructured region [30]. This ALR region binds to the CPC motif of MIA40 by substrate mimicry and re-oxidizes MIA40, resetting MIA40 into the native and active state, ready for another round of import [30]. The N-terminal CXXC motif (ALR sequence CRAC) then transfers electrons to the second CXXC motif (ALR sequence CEEC) of the ALR partner by inter-subunit electron transfer [29,30]. Finally, the electrons are transferred to a non-covalently bound FAD cofactor, which is near the CEEC sequence, and finally to a last electron acceptor such as molecular oxygen or cytochrome c [16,31]. Recently, upregulation and high protein levels of ALR have been linked to several forms of cancer, specifically to hepatocellular carcinoma, and a study about the inhibition of ALR showed drastic cell growth reduction related to the disruption of cellular iron levels (specifically heme iron) and decrease of complex I function [32]. To aid in mechanistic studies and to develop probes for modulating the MIA pathway in model systems, we conducted a small molecule screen to identify compounds that can bind to ALR and modulate the MIA pathway [33].

The molecules discovered in these experiments are referred to as MitoBloCK compounds, abbreviated as MB, originating from the Carla Koehler laboratory. In this investigation, we conducted a comprehensive analysis of the interactions between different MB compounds and ALR, utilizing 2D NMR (^1^H–^15^N HSQC) spectroscopy. Our primary goal was to determine which of these inhibitors binds to ALR and, on a structural level, to pinpoint the precise binding site on ALR. We further employed 2D NMR techniques to examine, at the atomic level, how the presence of the inhibitor bound to ALR affects its interaction with MIA40. We conducted titration experiments with the inhibited form of ALR and MIA40 to ascertain whether the various inhibitors share the same binding site on ALR.

## 2. Results

### 2.1. MB-6 Interaction with ALR

To rationalize the inhibitory activity of MitoBloCK-6 (MB-6) towards ALR, a description of the interaction at the atomic level is crucial. Two different NMR spectroscopy titrations of ^15^N-labeled ALR were performed: (i) on the full-length protein ALR (FL-ALR, 205 aminoacids, residues 1–205) and (ii) on a short form of ALR (SF-ALR, 125 aminoacids, residues 81–205). The SF-ALR construct is 125 residues long without the unstructured N-terminal domain that harbors the shuttle CXXC (CRAC) motif involved in the electron transfer from MIA40 to the CXXC (CEEC) motif in the ALR active site [30].

Upon addition of MB-6 to the ^15^N-labeled LF-ALR and SF-ALR, we were able to map all chemical shift changes in both titrations. The chemical shift changes observed are all clustered on the structured FAD binding domain for both protein constructs, and no chemical shifts changes were detectable on the unstructured N-terminal segment (residues 1–80) (see Supplementary Figure S1). Since the FL-ALR spectra exhibit a large overlap of NMR signals, mostly due to unstructured N-terminal domains, the chemical shift variations, i.e., those ascribed to the structured part of the protein of SF-ALR, were identical in the two titrations, and due to the relative instability of the FL-ALR construct (possibly due to the unstructured N-terminal domain) we performed the characterization of the inhibitors on the more stable SF-ALR for which the chemical shifts changes are mapped. The use of only SF-ALR is also supported by the chemical screen design that employed a non-physiological DTT substrate, which may have targeted the CXXC near the active site for oxidation [33].

**Figure 1:**
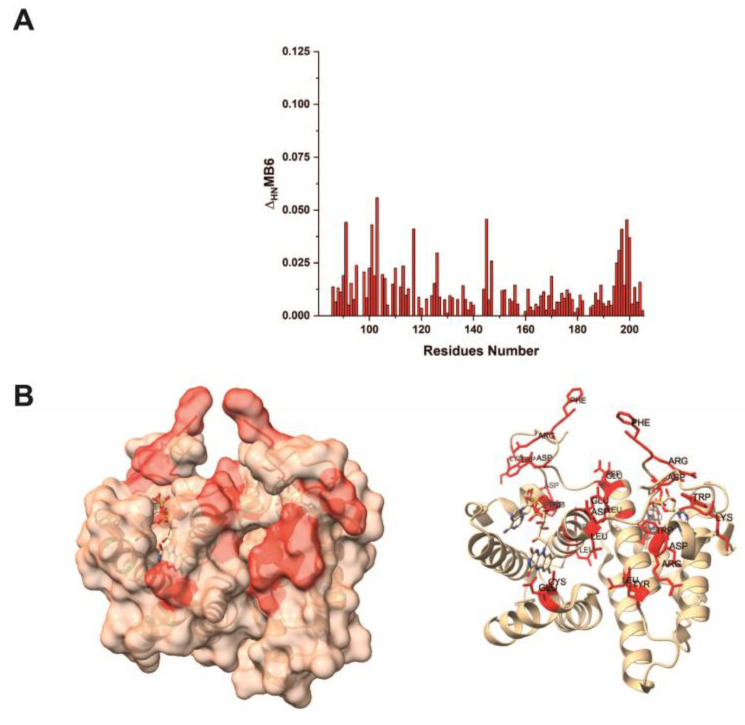
NMR analysis of the interaction between SF-ALR and MB-6. (**A**) Backbone weighted-average chemical shift differences Δ_HN_ for SF-ALR residues, observed upon addition of MB-6. (**B**) Dimeric SF-ALR mapped and labeled in red on the surface (represented in transparency); the FAD moiety is also depicted in both representations.

The ^1^H–^15^N HSQC spectra of SF-ALR (0.2 mM) with increasing amounts of MB-6 (up to 2 mM) showed the formation of a new species with increased signal intensities that correlated with the increased MB-6 concentration, whereas the signals of the free protein decreased until their disappearance. The residues of SF-ALR affected by the addition of MB-6 (Figure 1A), mapped on the ALR structure (PDB ID code 3O55) (Figure 1B), identified a well-defined region involving residues F91, R92, D94, D96, E101, L102, G103, L113, Y116, E144, C145, W195, R196, G197, W199, and K200 of SF-ALR. This region was then used to define structural models of protein–compound complexes using the HADDOCK 2.4 software.

In the calculations with HADDOCK, the C2 symmetry of the ALR structure was also considered and two small molecules per dimer of SF-ALR were docked. For MB-6, docking calculations clustered 176 structures in two separate clusters out of the 200 calculated structural models, which represented 88.0% of the water-refined models generated by HADDOCK. The statistical analysis of the two clusters is reported in Table 1. Both clusters show a relatively extended Buried Surface Area, indicating extensive interaction and a good overall RMSD within the members of each cluster. The major contribution to the interaction score originates in both clusters from the Van der Waals and Buried Surface Area terms. The main difference between the two clusters is the number of structures (137 vs. 39) and the contribution to the HADDOCK score of the electrostatic term (−19.7 ± 4.3 vs. −8.0 ± 13.3).

In summary, MB-6 is positioned in a hydrophobic pocket close to the CEEC motif of SF-ALR. This motif is essential for electron transfer from MIA40 to the FAD molecule bound to SF-ALR through cysteine pairs. The main hydrophobic contacts between MB-6 and each SF-ALR subunit of the dimer involve the aromatic rings of MB-6 and some hydrophobic residues at the interface of the SF-ALR dimer (L102 from one protein subunit and Y116, W195, W199 from the other protein subunit).

### 2.2. Additional MitoBloCK Compounds from ALR Screens

**Figure 2:**
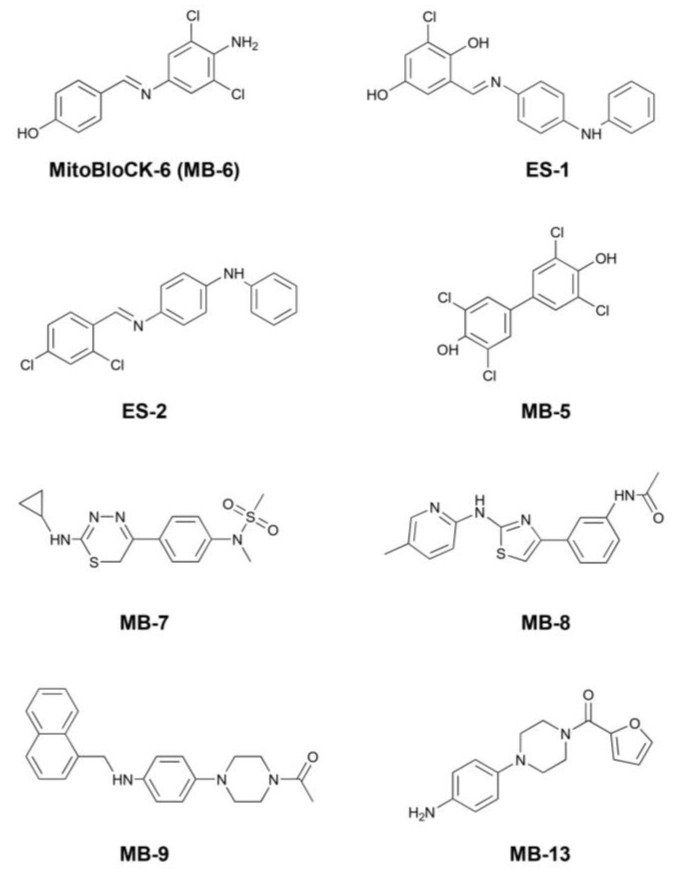
Structures of compounds that modulate Erv1 activity. MB-5 and 7 came from a previous screen and MB-8, -9, and -13 were from a collaborative screen.

Based on the interaction between MB-6 and SF-ALR, we investigated ALR interactions with additional small molecules that were identified in two chemical screens. Erv1 analogs ES-1 and ES-2 and MB-5 and MB-7 were identified in the initial Erv1 screen [33], whereas a second screen completed by the Broad Institute via the Molecular Libraries Probe Centers Network (MPLCN) yielded MB-8, MB-9, and MB-13 (Figure 2). The properties of MB-8, MB-9 and MB-13 were studied in vitro and in cellulo to assess toxicity and inhibition ability.

All three new compounds were able to inhibit ALR in a pure enzyme assay with an IC_50_ of 9.02 µM, 2.15 µM and 10.7 µM, respectively. MB-9 and MB-13 showed no toxicity in yeast and the HeLa cell when incubated with the two inhibitors at 200 µM and 100 µM, respectively. MB-8 presented a different toxicity profile based on the cell type. No toxicity was observed in yeast cells (at 200 µM), but MB-8 showed a toxicity effect in HeLa cells at the high concentration of 100 µM.

A detailed characterization of the interaction of this set of MB compounds (ES-1, ES-2, MB-5, MB-7, MB-8, MB-9, and MB-13) with SF-ALR was then performed. The small compounds can be chemically clustered into two subsets: one (Subset A), which includes ES-1, MB-5, and MB-8, characterized by partially charged aromatic rings (due to resonance forms) and no aliphatic elements; and the other (Subset B), including ES-2, MB-7, MB-9 and MB-13, in which the molecules have different heteroaromatic rings and polar terminals.

**Figure 3:**
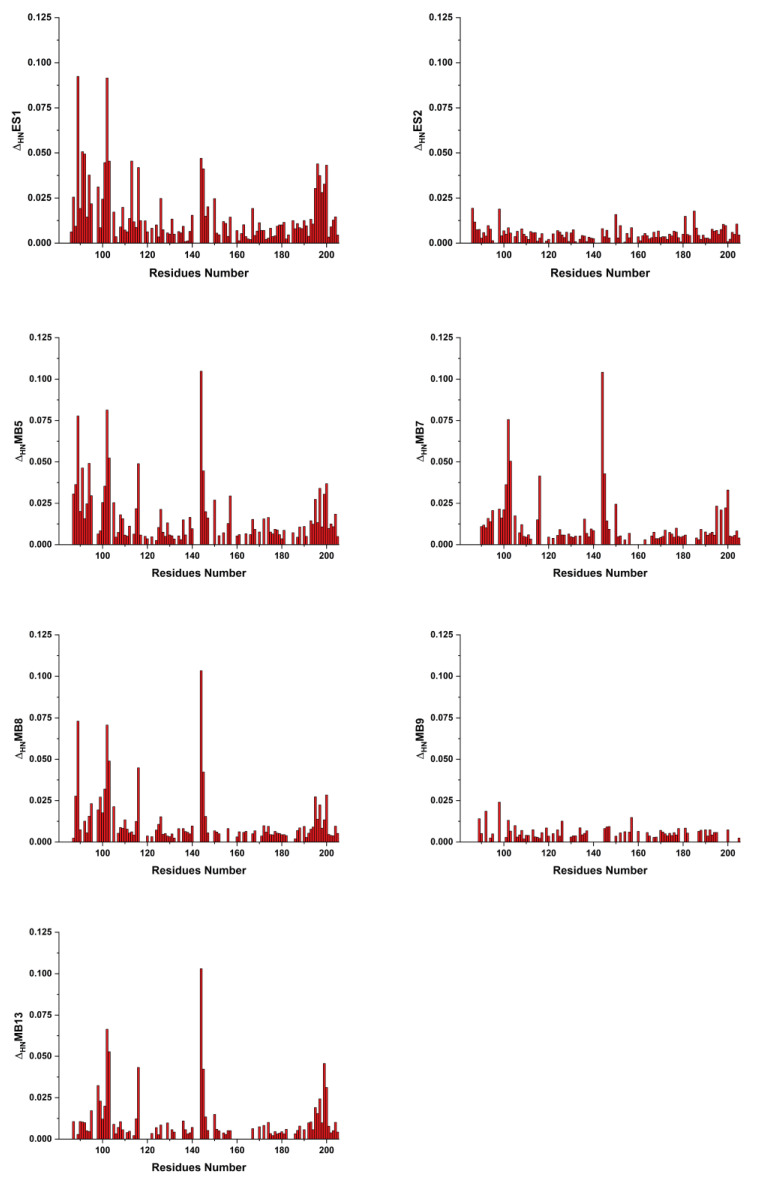
Mapping of the interaction between SF-ALR and MB compounds. The weighted-average chemical shift differences Δ_HN_ for SF-ALR residues upon addition of a small molecule (up to 2 mM for ES-1, ES-2, ES-9 and up to 4 mM for MB-5, MB-7, MB-8, and MB-13) are shown.

^1^H–^15^N HSQC spectra were acquired on SF-ALR (0.2 mM) with increasing amounts of each of the small molecules (up to 2 mM for ES-1, ES-2, ES-9 and up to 4 mM for MB-5, MB-7, MB-8, and MB-13), based on the available mass of each inhibitor, max 10% of DMSO, also present in solution. Two compounds (ES-2 and MB-9) did not show any interaction with ALR as indicated by no spectral changes up to ten-fold the concentration of the small molecule (Figure 3). For all these small molecules, as reported above for MB-6, the interaction residues are clustered on the structured domain of ALR, so the best candidate for the interaction study is SF-ALR since it comprises all the interacting residues and does not have stability issues in vitro. The interaction pattern is the same as the one reported for MB-6; indeed the interacting residues of SF-ALR are F91, R92, D94, D96, E101, L102, G103, L113, Y116, E144, C145, W195, R196, G197, W199, and K200 from each side of the two monomers within the homodimeric structure.

**Figure 4:**
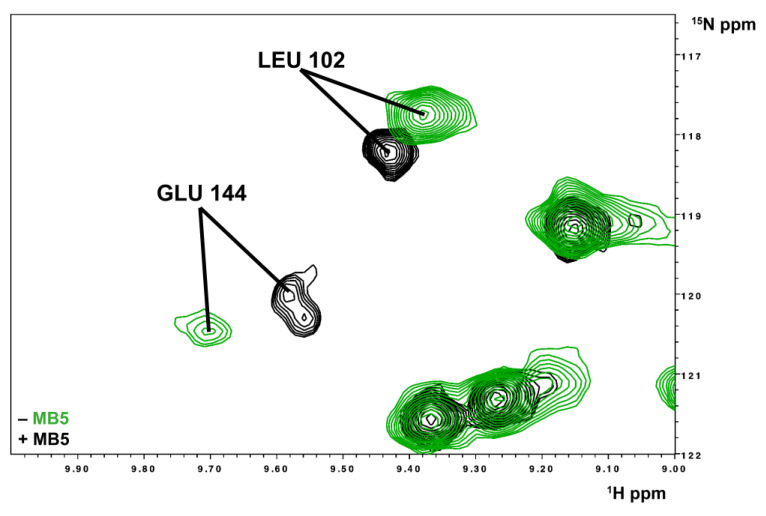
Interaction between MB-5 and SF-ALR. Overlay of ^1^H–^15^N HSQC spectral regions of ^15^N-labeled SF-ALR unbound (green) and SF-ALR bound to MB-5 (black) in protein:ligand ratio 1:10.

For all interacting molecules, the compound-bound form of SF-ALR is in slow exchange (on the NMR time scale, i.e., slower than 10^−2^ s) with the free ones. Indeed, upon addition of increasing amounts of a specific compound (any of the ones considered in the present study), the formation of a new species in the NMR spectra of the protein was detected, and its signal intensities were found to increase upon increasing the concentration of the compound, while the signals of the unbound species disappeared (Figure 4).

This behavior suggests a typical tight binding as a slow exchange regime is often observed for high-affinity interactions. The two subsets of the small molecules have specific interaction patterns with SF-ALR. Those belonging to specific Subset A interact with residues of ALR located at the dimer interface of the protein as in the case of MB-6 interaction. The second subset of molecules interacts only with a restricted number of residues, i.e., Y76, E144, C145, W195, R196, G197, W199, and K200.

The best cluster statistical analysis for each structural model calculated for each of the analyzed compounds (ES-1, MB-5, MB-7, MB-8, and MB-13) is reported in Table 2. The analysis follows the same scheme as for MB-6. Only the complex with MB-8 showed a distribution in two clusters, while with all the other compounds, essentially only one cluster was obtained. The investigation also showed that all these compounds have comparable values for all the evaluated HADDOCK parameters in the docking calculations.

**Figure 5:**
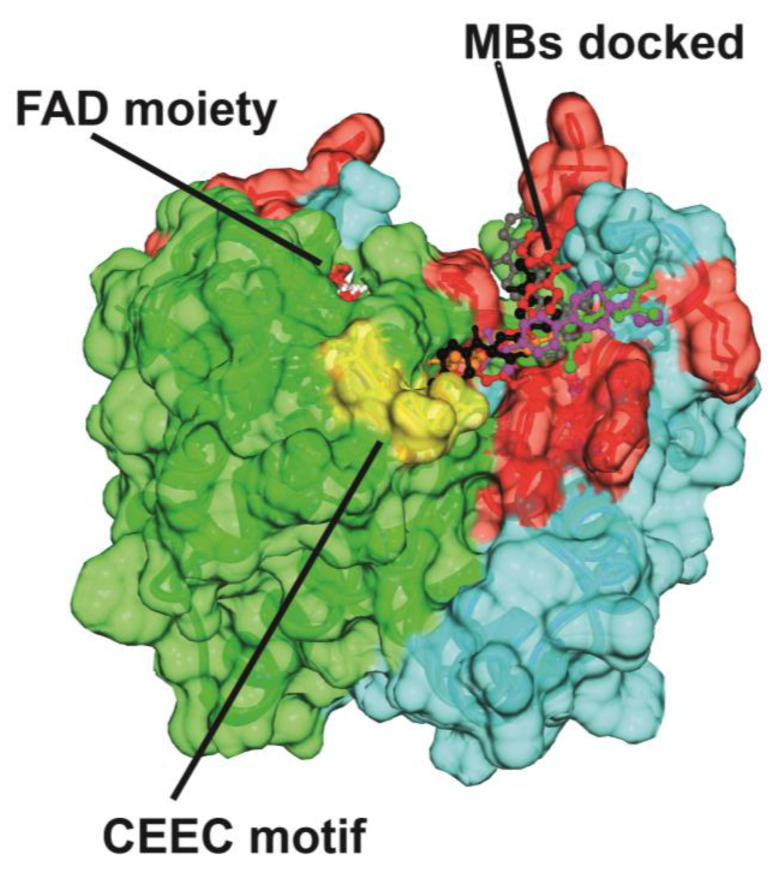
Adduct between dimeric SF-ALR and all the compounds. The surfaces of the two monomers of SF-ALR (green protein Subunit A and cyan protein Subunit B) are shown and all the compounds are highlighted with different colors in ball-and-stick representation. The CEEC motif is shown as yellow transparency, and the FAD moiety is indicated in ball-and-stick representation. The interacting residues are shown in transparency in red under the surface.

The overlap of all calculated structural models of the adducts between the dimeric SF-ALR with each of the compounds tested (Figure 5) shows that all the compounds have the same mode of interaction and mostly sit in the same protein region, i.e., the segment that comprises residues 81–205 of SF-ALR. Overall, the computational comparison of all the tested compounds showed that the optimal inhibitor for this system is MB-7. Indeed, MB-7 shows the best HADDOCK score, RMSD from the overall lowest-energy structure, Electrostatic energy, Desolvation energy, Buried Surface Area compared to all the other tested compounds.

### 2.3. MIA40 and Small Molecules Bind to Separate Regions of ALR

Based on how the small molecules interact with ALR, they all sit in the proximity of the CEEC motif; potential mechanisms that impair steps in the electron transfer process in the MIA40/ALR complex may center on the CEEC motif, near to the FAD prosthetic group. Specifically, electron transfer might be affected either from the CRAC to the CEEC motifs or from the CEEC motif to FAD. The fact that the interaction pattern is the same for the full-length protein (LF-ALR) and the short construct (SF-ALR) indicates that the analyzed small molecules might not affect the interaction between ALR and MIA40. To further investigate a possible interference of the small molecules on the ALR-MIA40 interaction, the CRAC-ALR construct (SF-ALR plus 20 residues of N-terminal domain, residues 61–205 of FL-ALR) was used as it contains the protein region interacting with MIA40, i.e., the flexible N-terminal domain of ALR.

This construct, when titrated with MB-5, gave rise to the same spectral changes (i.e., structured region), thus indicating that the additional 20 residues present in this construct are not involved in MB-5 binding. The 1:1 CRAC-ALR/MB-5 adduct was then titrated with MIA40. MIA40 still interacts with the 20 residues of the flexible N-terminal domain of ALR, but no further spectral changes were observed on the C terminal domain. This confirms that MIA40 and the small molecules interact with two different sites of ALR: (i) MIA40 with residues located at the flexible N-terminal domain; (ii) the small molecules with the accessible hydrophobic cleft in ALR. These two sites of ALR are not linked with each other in this case, but they are subject to transient interaction when electron transfer is active [30]. To confirm the specificity of MB compounds against ALR, ^15^N-labeled MIA40 was titrated with the MBs used in this study and no chemical shift change was observed, proving that the small molecules do not interact with MIA40.

## 3. Discussion

MB compounds represent a class of molecules that have been identified through several screening and research efforts to target mitochondrial processes, especially those involving ALR in the IMS. Their discovery might have implications for understanding mitochondrial biology, exploring potential treatments for mitochondrial diseases, cancer, neurodegenerative diseases and advancing drug development in these fields.

Here, we report the first structural characterization of a collection of small molecules that target the sulfhydryl oxidase ALR in mitochondria. From our small molecule screens, we find that the small molecules can target yeast and mammalian proteins equally well [33,34,35,36], likely because the overall structures of import proteins are highly conserved throughout the evolution of the ALR protein. Thus, an optimal collection of probes that are useful for studying the MIA pathway mechanisms and function in different systems is available.

### Structure Function Studies

The small molecules that induce a perturbation on the HN signals in the 2D NMR spectra (ES-1, MB-5, MB-7, MB-8, and MB-13), although diverse in structure, target a very similar region in ALR. Most likely, this is due to a combination of hydrophobic and polar regions on the ligand. The structural diversity could be also useful in the future synthesis of second-generation ALR inhibitors since different moieties and heteroaromatic rings could be used in the development of other more potent and more specific compounds with different pharmacodynamic and pharmacokinetic profiles. Common structural features for the interaction are here reported: all the MB molecules bind in a hydrophobic pocket close to the CEEC motif, essential for mediating electron transfer from MIA40 through the two cysteine pairs (CRAC motif) to FAD bound to ALR; indeed, the CEEC motif sits close to the FAD moiety. The interaction patterns that can favor the binding of these small molecules are the hydrophobic contacts such as van der Waals contacts and π–π stacking interactions. Indeed, the main hydrophobic contacts between the small molecules and ALR (two per ALR dimer) involve the aromatic rings of MBs and hydrophobic and aromatic residues such as F91, L102, G103, L113, Y116, W195, G197, and W199 at the interface of the ALR dimer. Out of the whole collection, MB-7 seems to have the highest specificity.

**Figure 6:**
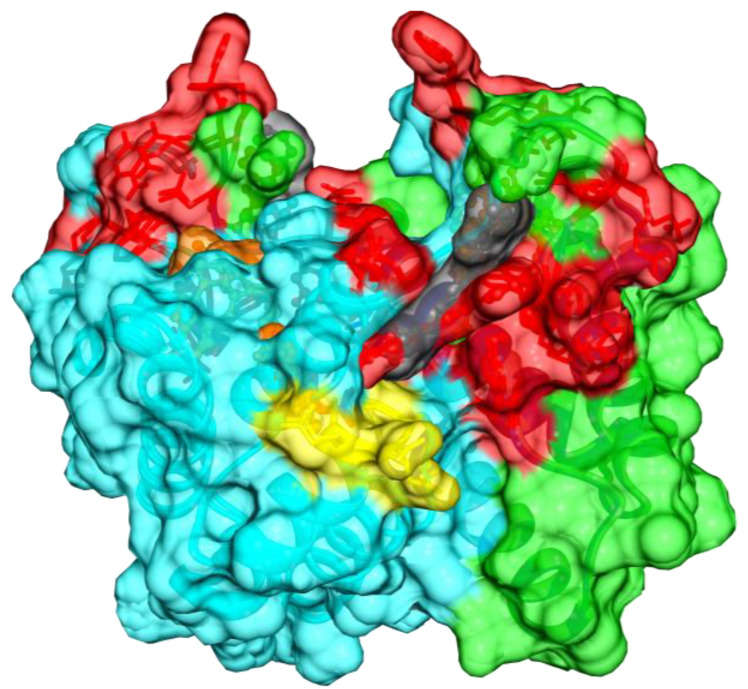
Adduct between dimeric SF-ALR with MB-6 and MB-7. The surfaces of the two monomers of SF-ALR (green protein Subunit A and cyan protein Subunit B) are shown, and MB-6 (red) and MB-7 (black) compounds are highlighted in ball-and-stick representation. The CEEC motif is shown as yellow transparency, and the FAD moiety is indicated in ball-and-stick representation. The interacting residues are shown in transparency in red under the surface.

Specifically, MB-6 and MB-7 have different heteroaromatic rings and polar terminal groups, but they have a similar spatial distribution of hydrophobicity and polarity. Nonetheless, sitting in almost the same area in the hydrophobic cleft of ALR, the main difference on the mode of interaction between MB-6 and MB-7 resides in the fact that the opposite terminals (hydrophobic) have different orientations; the MB-6 hydrophobic terminal is pointing towards the intermolecular dimer surface close to the intermolecular disulfide bridge; instead, the MB-7 hydrophobic terminal points to the direction of the last loop of one of the monomers (Figure 6). From a structural point of view, this atomic information can be exploited to design new compounds. This can lead us to speculate on the different effects on the MIA-dependent import pathway.

The different ability of the MBs to inhibit import might be rooted in the MIA40/ALR electron transfer mechanism rather than inhibiting interaction between MIA40 and ALR. For example, MB-7 might block electron transfer between the CRAC motif (in the N terminal domain) and the CEEC motif (in the folded, C terminal domain), somehow preventing MIA40 from binding to a substrate. In contrast, MB-6 might impair electron transfer between the CEEC motif and the riboflavin ring of FAD, preventing MIA40 from releasing the substrate. Alternatively, the small molecules may interfere with the formation of the MIA40-ALR complex or a ternary complex with substrates in mitochondria [37,38], which can only be observed in intact ones. Summarizing, this initial structure–function study yields a collection of ALR-specific probes that can be used for detailed mechanistic studies, particularly focusing on disulfide redox exchange [33,39].

The MIA pathway is thus becoming important as a drug target in mammalian systems. ALR plays a critical role in stem cell pluripotency [33] and the MIA pathway is required for the import of substrates such as CHCHD2 and CHCHD10 that are important in neurodegeneration [23].

Given that our MBs might cause the arrest of precursors at different stages in translocation, they represent new probes to aid in biogenesis studies for these substrates, enriching the development of disease models [33]. Thus, our initial structure–function studies have generated a new collection of specific probes for ALR, which bind to a region that is critical for catalysis. These probes are useful for further refinement of the disulfide relay mechanism and for the development of new models for studying degenerative diseases.

In summary, here, NMR spectroscopy provided a method to investigate protein–ligand interactions in solution, mimicking, even though not fully reproducing, the physiological flexibility of a protein target present in the cell compartment. Appropriate protein preparation protocols coupled with sensitive NMR experiments and systematic data analyzing schemes were applied for a drug discovery investigation that clearly showed that the integration of several techniques is needed. There is, in any case, a limitation in this study. Characterization was carried out mainly in vitro and in silico with some information on the inhibitory activity in cellulo (see IC_50_) by Koehler and coworkers [33,39]. To further validate the hypothesis of the mechanism of the inhibition of ALR activity, a more extensive drug discovery investigation is necessary that targets more specifically the CEEC motif and the FAD electron transfer mechanism.

Overall, here it was shown that MB compounds discovered in the ALR screens through NMR spectroscopy in combination with previously reported studies hold promise for advancing our understanding of mitochondrial biology and may have therapeutic implications for diseases related to mitochondrial dysfunction. The successful development, application and optimization of these compounds depend on further research, such as in vivo studies.

## 4. Materials and Methods

### 4.1. Protein Expression and Purification

LF-ALR, SF-ALR, CRAC-ALR and MIA40 were expressed and purified following previously reported conditions; briefly, the genes of the three ALR constructs (LF-ALR, SF-ALR and CRAC-ALR) and MIA40 were cloned using the Gateway^®^ system into pDEST-His-MBP (Addgene, Teddington, UK) to generate N-terminal His-MBP fused proteins. The three ALR constructs were expressed in *Escherichia coli* BL21 (DE3) gold cells (Stratagene, Amsterdam, Netherlands), which were grown in Luria–Bertani (LB) or a minimal medium containing [^15^N]-(NH_4_)_2_SO_4_ to produce labeled samples. Protein expression was induced with 0.4 mM IPTG for 16 h at 20 °C [15]. To guarantee the presence of the FAD cofactor, a vitamin mix was added in both LB and minimal media [15,27].

MIA40 was expressed in *Escherichia coli* Origami pLysS cells (Novagene, Cambridge, UK), in Luria-Bertani or in minimal medium [^15^N]-(NH_4_)_2_SO_4_ to produce labeled samples. MIA40 expression was induced with 0.7 mM IPTG for 16 h at 25 °C [14,30,33]. For all proteins, the cell pellet was resuspended in a binding buffer (50 mM Tris-HCl, pH 8 containing 500 mM NaCl, 5 mM imidazole, 0.01 mg/mL DNAase, 0.01 mg/mL lysozyme, 1 mM MgSO_4_ and 5 mM DTT) and sonicated in ice. Purification was performed using a HiTrap chelating HP column (GE Healthcare, Milano, Italy) charged with Ni(II), and the recombinant proteins were eluted with 50 mM Tris-HCl (pH 8), 500 mM NaCl and 500 mM imidazole. The His-MBP tag was cleaved with TEV protease in 50 mM Tris-HCl (pH 8), 500 mM NaCl, 5 mM imidazole and 3 mM DTT at room temperature overnight. The cleaved proteins were separated from the protein tag using a second HiTrap chelating HP column. A purification step was then performed using a HiLoad 16/60 Superdex 75 prep grade (Amersham Pharmacia Biosciences, GE Healthcare, Milano, Italy) gel filtration column. All the purification steps were followed by SDS-PAGE gel analysis [14]. Protein concentration was determined by its absorption at 280 nm using an extinction coefficient of 26,970 M^−1^ cm^−1^ for SF-ALR, 31,970 M^−1^ cm^−1^ for all the other ALR constructs and 13,325 M^−1^ cm^−1^ for MIA40.

### 4.2. Titration Studies on ALR

^1^H–^15^N HSQC NMR spectra were recorded to follow the interaction between the small molecules and various constructs of ALR. A 0.2 mM solution of both ALR constructs in 50 mM phosphate, 0.5 mM EDTA pH 7.0 was titrated with all the inhibitors dissolved in DMSO (up to 2 mM for ES-1, ES-2, ES-9 and up to 4 mM for MB-5, MB-7, MB-8, and MB-13). Each inhibitor was added to a diluted solution of ALR (5 mL of 50 mM phosphate pH 7.0), incubated at room temperature for 15 min, then concentrated to 450 µL using a 5 K cutoff Centricon^®^(Merk Life Science s.r.l., Milano, Italy). A total of 50 µL of D_2_O were added, and then the sample was transferred into the NMR tube. This dilution–concentration approach was chosen to minimize the solvent effect of DMSO on the protein sample. ^1^H–^15^N HSQC NMR spectra were recorded on a Bruker AVANCE 500 MHz at 308 K equipped with a cryoprobe.

The NMR spectrum of the sample containing ALR alone was tested with an addition of DMSO corresponding to the final amount of DMSO at the end of the titration in order to exclude any spectral change induced by DMSO (10% DMSO in the final samples that correspond to about a 20 μM concentration). The initial spectrum was then acquired with only the ALR protein for each of the different constructs. These spectra served as the baseline for the following incremental additions. After each addition, the NMR spectrum of the mixture was recorded. This process was repeated multiple times, with the titrant concentration increasing in each step, reaching an excess of ligands (MBs). Spectral changes were also monitored during the titration, looking for spectral changes including chemical shift perturbations (shifts in resonance frequencies), changes in peak intensities or line shapes, and broadening of resonances.

### 4.3. Titration Studies of ALR:MB Molecules with MIA40

^1^H–^15^N HSQC NMR spectra were recorded to follow the interaction between the ^15^N-labeled ALR:MB complexes with MIA40. The ALR:MB complexes were prepared following the same procedures as reported in Section 4.2. A 0.2 mM solution of the CRAC ALR construct (residues 60–205) with MB already present in a 50 mM phosphate, a 0.5 mM EDTA, pH 7.0, was titrated with unlabeled MIA40 (up to a 1:1 ratio was reached). ^1^H-^15^N HSQC NMR spectra were recorded on Bruker AVANCE 500 MHz at 308 K equipped with a cryoprobe.

This NMR method (^1^H–^15^N HSQC spectra) was also used for titration of ^15^N-labeled MIA40 with small molecules to exclude the interaction of these small molecules with MIA40.

### 4.4. Chemical Shit Perturbation Analysis

All chemical shift perturbation analyses were performed using the weighted-average chemical shift differences Δ_HN_, that is, ([(Δ_H_)^2^ + (Δ_N_/5)^2^]/2)^1/2^), where Δ_H_ and Δ_N_ are chemical shift differences for ^1^H and ^15^N of ALR in the presence of MB molecules before and after their addition. All chemical shifts used for the starting point of ALR constructs and MIA40 were extracted from NMR assignments previously deposited in the BMRB database (codes: 15763, 18360, 18631, 18029).

### 4.5. Inhibition Analysis via Chemiluminescence Assay

The reported IC_50_ values were provided by Dr. Koehler’s laboratory, where the test was set up using an in vitro Amplex Red-HRP assay. This method was extensively reported by Dabir et al. [33,39].

### 4.6. Docking Calculations

Structural models for the complex between human SF-ALR and all the interacting compounds were obtained with the HADDOCK program [40,41], combining chemical shift perturbation analyses and in silico docking. In the calculations, the C2 symmetry of the ALR structure was considered and two small molecules per dimer of SF-ALR were docked. The structures of human SF-ALR (PDB ID 3O55) and the structures of the compounds, generated with PRODRG software, were used as input, respectively. The entire docking protocol, already established by Bonvin and coworkers, in HADDOCK consists of five stages: (i) generation of topologies and structures with the input of PRODRG for the small molecules, (ii) generation of topologies and starting coordinates for the complex, (iii) randomization of starting orientations and rigid-body energy minimization, (iv) semi-flexible simulated annealing, and (v) flexible final refinement.

## Figures and Tables

**Table 1:** Calculated parameters of the data-driven docking of MB-6 to SF-ALR.

HADDOCK Parameters	Cluster 1	Cluster 2
HADDOCK score	−19.8 ± 0.5	−14.0 ± 1.9
Cluster Size (Number of structures)	137	39
RMSD from the overall lowest-energy structure (Å)	0.4 ± 0.2	0.6 ± 0.0
Van der Waals Energy	−44.1 ± 3.1	−47.6 ± 2.4
Electrostatic Energy	−19.7 ± 4.3	−8.0 ± 13.3
Desolvation Energy	15.9 ± 5.3	22.8 ± 7.1
Restraints Violation Energy	118.6 ± 34.8	119.3 ± 44.5
Buried Surface Area (Å^2^)	1140.2 ± 31.9	1182.1 ± 24.6
Z-Score	−1.2	−0.1

**Table 2:** Calculated parameters of the data-driven docking of all analyzed MBs to SF-ALR. Note that ES-2 and MB-9 are not listed here since NMR does not show any interaction.

HADDOCK Parameters	First Set of Molecules				Second Set of Molecules	
	ES-1	MB-5	MB-8 (1)	MB-8 (2)	MB-7	MB-13
H Score	−16.4 ± 2.6	−14.1 ± 4.1	−41.8 ± 3.1	−31.6 ± 2.6	−47.8 ± 2.4	−40.2 ± 3.7
CS	177	154	134	45	156	166
RMSD	0.4 ± 0.2	0.4 ± 0.2	0.4 ± 0.2	0.5 ± 0.1	0.4 ± 0.2	0.4 ± 0.2
VdW En	−43.0 ± 0.9	−37.6 ± 0.8	−45.9 ± 5.6	−42.5 ± 3.7	−37.3 ± 3.8	−45.7 ± 2.8
Elec En	−15.7 ± 10.1	−1.5 ± 1.9	−56.9 ± 19.1	−52.8 ± 10.0	−191.2 ± 20.5	−43.8 ± 15.5
Des En	19.4 ± 2.7	9.7 ± 2.2	11.6 ± 4.9	15.8 ± 2.3	19.6 ± 2.4	11.5 ± 3.3
RV En	98.8 ± 38.48	135.2 ± 38.12	33.5 ± 46.07	53.2 ± 25.00	77.0 ± 16.99	24.1 ± 19.42
BSA	953.0 ± 27.4	962.5 ± 18.8	1069.8 ± 74.8	1057.6 ± 38.1	1088.7 ± 23.6	944.4 ± 56.3
Z-score	−1.4	−1.3	−1.4	0.3	−1.7	−1.7

## Data Availability

Data available upon request.

## Supplementary Materials

The following supporting information can be downloaded at: https://www.mdpi.com/article/10.3390/ijms25021174/s1.
