# Supplementary material for: The Interaction and Effect of a Small MitoBlock Library as Inhibitor of ALR Protein–Protein Interaction Pathway
Source: Int J Mol Sci. 2024 Jan 18;25(2):1174. doi: 10.3390/ijms25021174 (PMC10816046; doi:10.3390/ijms25021174)
Supplement: Supplementary file 1 [file ijms-25-01174-s001.zip › ijms-2797853-supplementary.pdf]

Supplementary Figure S1

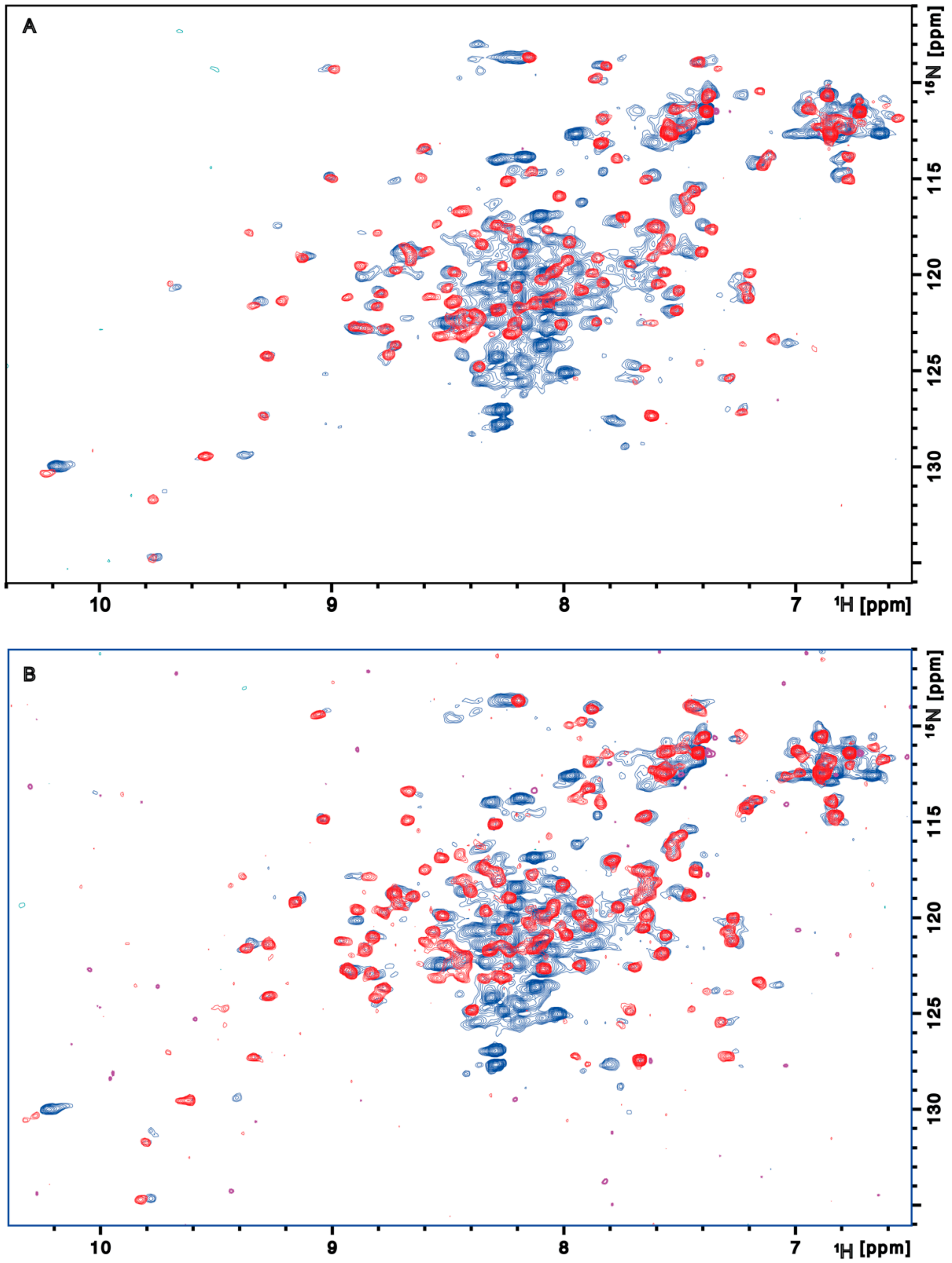

Figure S1. (**A**) ^{1}H–^{15}N HSQC overlap of LF-ALR (residues 1–205; blue) and SF-ALR (residues 81-205; red); (**B**) ^{1}H–^{15}N HSQC overlap of LF-ALR in presence of MB6 (residues 1–205; blue) and SF-ALR in presence of MB6 (residues 81–205; red). To be noted that the chemical shifts difference of the protein in their native states are the same in the MB6 bound proteins.
